# Paraoxonase 1 Polymorphism and Prenatal Pesticide Exposure Associated with Adverse Cardiovascular Risk Profiles at School Age

**DOI:** 10.1371/journal.pone.0036830

**Published:** 2012-05-15

**Authors:** Helle R. Andersen, Christine Wohlfahrt-Veje, Christine Dalgård, Lene Christiansen, Katharina M. Main, Christine Nellemann, Katsuyuki Murata, Tina K. Jensen, Niels E. Skakkebæk, Philippe Grandjean

**Affiliations:** 1 Institute of Public Health, University of Southern Denmark, Odense, Denmark; 2 University Department of Growth and Reproduction, Rigshospitalet, Copenhagen, Denmark; 3 National Food Institute, Division of Toxicology and Risk Assessment, Technical University of Denmark, Søborg, Denmark; 4 Department of Environmental Health Sciences, Akita University Graduate School of Medicine, Akita, Japan; 5 Harvard School of Public Health, Boston, Massachusetts, United States of America; University of Montreal, Canada

## Abstract

**Background:**

Prenatal environmental factors might influence the risk of developing cardiovascular disease later in life. The HDL-associated enzyme paraoxonase 1 (PON1) has anti-oxidative functions that may protect against atherosclerosis. It also hydrolyzes many substrates, including organophosphate pesticides. A common polymorphism, *PON1* Q192R, affects both properties, but a potential interaction between *PON1* genotype and pesticide exposure on cardiovascular risk factors has not been investigated. We explored if the *PON1* Q192R genotype affects cardiovascular risk factors in school-age children prenatally exposed to pesticides.

**Methods:**

Pregnant greenhouse-workers were categorized as high, medium, or not exposed to pesticides. Their children underwent a standardized examination at age 6-to-11 years, where blood pressure, skin folds, and other anthropometric parameters were measured. *PON1*-genotype was determined for 141 children (88 pesticide exposed and 53 unexposed). Serum was analyzed for insulin-like growth factor I (IGF-I), insulin-like growth factor binding protein 3 (IGFBP3), insulin and leptin. Body fat percentage was calculated from skin fold thicknesses. BMI results were converted to age and sex specific Z-scores.

**Results:**

Prenatally pesticide exposed children carrying the *PON1* 192R-allele had higher abdominal circumference, body fat content, BMI Z-scores, blood pressure, and serum concentrations of leptin and IGF-I at school age than unexposed children. The effects were related to the prenatal exposure level. For children with the *PON1* 192QQ genotype, none of the variables was affected by prenatal pesticide exposure.

**Conclusion:**

Our results indicate a gene-environment interaction between prenatal pesticide exposure and the *PON1* gene. Only exposed children with the R-allele developed adverse cardiovascular risk profiles thought to be associated with the R-allele.

## Introduction

The serum enzyme paraoxonase 1 (PON1) is a high density lipoprotein (HDL)-associated enzyme with antioxidant function [1]. It protects lipoproteins from oxidative modifications, and thus protects against the development of atherosclerosis [2], [3]. It also catalyzes the hydrolysis of a wide range of substrates including the toxic oxon metabolites of several organophosphates and therefore provides some protection against chronic exposure to these pesticides [4], [5].

Several polymorphisms in *PON1* have been identified [6], [7]. A common polymorphism in the coding sequence, a glutamine (Q)/arginine(R) substitution at position 192, affects the catalytic capacity toward pesticides [5], [8], and also the anti-atherogenic properties [2]. Hence, an association between the R-allele and the risk of developing cardiovascular disease (CVD) has been reported in case-control studies [2], [9], [10]. The ability of *PON1* to protect against both organophosphate toxicity and atherosclerosis is also supported by experimental studies, since *PON1*-knockout mice were more sensitive to the toxic effects of chlorpyrifos, and developed atherosclerosis when fed a high-fat diet [11]. Although the *PON1* Q192R genotype seems to affect both the catalytic activity and the anti-atherogenic properties, the potential interaction between this genotype and pesticide exposures on cardiovascular risk factors has not been investigated.

We recently reported lower birth weight followed by an increased body fat accumulation from birth to school age in children whose mothers were exposed occupationally to modern non-persistent pesticides in early pregnancy [12]. In this cohort of children, we have determined the *PON1* genotype to investigate possible interactions between *PON1* Q192R polymorphism and prenatal pesticide exposure on risk markers for development of CVD later in life.

## Methods

### Study Population and Design

This study is a part of an ongoing prospective study of the effects of pesticide exposure in early pregnancy on the growth and development in the children. From 1996 to 2000 pregnant women working in greenhouses and referred to the Department of Occupational Health at Odense University Hospital in Denmark for advice regarding their working conditions during pregnancy, were recruited consecutively [13]. Their children were examined for the first time at three months of age [13] and followed up at school age [12].

At enrolment (gestational weeks 4–10), detailed information about working conditions, pesticide use and exposure was obtained from maternal interviews and supplemented by telephone contact to the employers. For all women, re-entry activities (such as moving or packing pot plants or nipping cuttings) constituted their main work functions. Approximately 20% of the women reported to have been directly involved in applying pesticides, mainly by irrigating fungicides or growth retardants. Only few (6%) of the women had applied insecticides.

The total pesticide exposure level was categorized independently (with agreement in all cases) by two toxicologists with special expertise in working conditions in greenhouse horticultures into three groups: none/low (controls), medium, and high as previously described [12], [14]. The exposure classification was performed before the first examination of children. Approximately 200 different pesticide formulations, representing 124 different active pesticide ingredients, were used in the working areas. Some were used only in few greenhouses or during restricted time periods, whereas, others were used more often. Three organophosphates: dichlorvos, dimethoate, and chlorpyriphos, were among the 20 most frequently used pesticides, and twelve other organophosphates were used less frequently. For most (91%) of the women rated as pesticide exposed, organophosphates had been used in the working area, but the time interval between applying insecticides and working in the treated areas was longer (1–3 days) than for fungicides and growth regulators (often a few hours). Hence, the re-entry exposure was regarded higher for fungicides and growth regulators than for insecticides. Women categorized as pesticide exposed, went on paid leave or were moved to work functions with less or no pesticide exposure shortly after enrollment. Hence, pesticide exposure mainly occurred early in pregnancy.

A total of 168 children were categorized as prenatally pesticide exposed, and 35 children were categorized as unexposed. Of these children 133 (65.5%) accepted an invitation to participate in a follow-up study when the children were between 6 and 11 years old. In this group 112 (59 boys and 53 girls) were prenatally pesticide exposed and 21 (14 boys and 7 girls) were unexposed. To supplement the unexposed group, the participating families were asked to invite children from family, friends and neighbours to participate. Only children between 6 and 11 years whose mother had not been occupationally exposed to pesticides during pregnancy could participate. Through this method, 44 children were recruited [12].

### Ethics

The study was conducted according to the Helsinki II Declaration with written informed consent by all mothers and was approved by The Regional Scientific Ethical Committee for Southern Denmark and the Danish Data Protection Agency.

### Questionnaire

All families completed a questionnaire prior to the examination of the child with information on medical history, education, occupation, living conditions, life style and diet. An additional short questionnaire regarding smoking, alcohol intake, medical use, disease and occupation during pregnancy was answered by the ‘new’ families for whom we did not have this information from the first examination. Information on gestational age at birth, birth weight and length was obtained from obstetric records.

Social class of the family was coded based on the parents education and occupation according to the criteria of the Danish National Institute of Social Research [15], [16], which is almost identical to the UK Registrar General’s classification of five social classes ranked 1 (high) to 5 (low). The social class of the higher ranked parent living with the child was used.

### Clinical Examination

The children underwent a standardized physical examination. Body weight (kg) was measured on a digital weight scale with a precision of 0.1 kg (TBF-300, Tanita Europe, UK). Standing height (cm) was measured to the nearest 0.1 cm using a transportable stadiometer (Chasmors LTD., London, UK).

Thickness of skin folds (mm) at triceps, subscapular, biceps and flank/suprailiac were measured by a caliper (John Bull, British indicators LTD, UK) with a precision of 0.1 mm after allowing the jaws to close on the fat fold for two seconds as described by Rodriguez et al [17]. Abdominal circumference was measured at the level of the umbilicus in a horizontal line with a tape measure to the nearest mm. All anthropometrical measurements were measured in triplicate and means were used for analysis.

Systolic and diastolic blood pressure was measured three times with an automated non-invasive blood pressure monitor (Colin Press-Mate, Colin® Corporation, Japan) and means were used for analysis.

Heart rate was measured on a Heart Rate Monitor (Polar s810) connected to a computer. After the child had been lying in a relaxed, supine position for at least 5 minutes, 300 R-R intervals were measured and 100 consecutive R-R intervals with the lowest standard deviation (SD) were extracted for calculation of the coefficient of variation for heart rate (CV_RR_). CV_RR_ is the ratio in (percent) of the SD of the R-R intervals to the mean R-R interval (SD*100/mean R-R interval). Due to technical problems with the Heart Rate Monitor during four of the examination days, heart rate was obtained only for 128 of the 177 children.

The same paediatrician (CWV) performed all clinical examinations blinded to information about maternal pesticide exposure during pregnancy.

Genotyping and all serum analyses were performed blinded to both exposure information and examination outcomes.

### Laboratory Analysis

Venous non-fasting blood samples were obtained (between midmorning and late afternoon) from 145 of the children. The samples were collected in EDTA coated and uncoated vials (Venoject). After centrifugation at 2000 g for 10 min at 20°C, buffy coat for genotyping was separated from the EDTA-treated samples. Buffy coat and serum from the uncoated vials were stored at −80°C until analysis.

### Metabolic Biomarkers

Insulin (proinsulin and insulin) and leptin concentrations in serum were analyzed using commercial hormone kits from RayBio (RayBio Human Insulin Elisa Kit or Human Leptin Elisa kit, cat. no. ELH-insulin-001 human and ELH-leptin-001, AH Diagnostics, Århus, Denmark). The assays were performed with immobilized specific antibody-coated 96-well plates. The amounts of both insulin and leptin were quantified using standard curves for each antigen evaluated in the same analysis (for details see the manufactureŕs instructions). Limits of detection were 6 pg/ml for leptin and 160 pg/ml for insulin and interassay variation was less than 10% and 12% for leptin and insulin, respectively.

Insulin-like growth factor 1 (IGF-I) and insulin-like growth factor binding protein 3 (IGFBP3) were measured in serum with solid-phase enzyme-labelled chemiluminescent immunometric assays (Immulite 2000; Diagnostic Products Corporation, Los Angeles, CA, USA) using World Health Organization (WHO) NIBSC IRR 87/518 and 93/560 standards, respectively. The limits of detection were 25 ng/ml and 500 ng/ml, with intra- and interassay variation less than 5 and 7%, respectively.

### Genotyping of *PON1*

DNA was isolated from buffy coats and C-108T (rs705379), M55L (rs854560) and Q192R (rs662) polymorphisms of the *PON1* gene were determined by the Taqman-based allele discrimination using the ABI Prism 7700 Sequence Detection System, as previously described [18]. *PON1* genotype was performed successfully for 141 of the children.

### PON1 Activity

The serum activity of PON1 was determined with paraoxon as substrate as previously described [19], [20]. Briefly, serum was added to Tris buffer (100 mmol/l, pH 8.0) containing 2 mmol/l CaCl_2_ and 5.5 mmol/l paraoxon (O,O-diethyl-O-p-nitrophenylphosphate, Sigma Chemical Co). The rate of p-nitrophenol generation was determined at 405 nm, 25°C, using a continuously recording spectrophotometer (Perkin Elmer, Lambda 11).

### Data Analysis

Since only 11 children had the RR genotype, those with the QR and RR type were combined in the data analysis.

Sum of four skinfolds (mm) was calculated as the sum of: triceps + subscapular + biceps + flank skinfolds (mm). Body fat percentage was calculated by a gender and age specific equation [21] based on triceps and sub scapular skin folds: For girls: (1.33(triceps+subscapular)- 0.013(triceps + subscapular)^2^ −2.5) when the sum of triceps and subscapular skinfold was lower than 35 mm, and (0.546(triceps+subscapular) +9.7) when the sum was higher than 35 mm. For boys: (1.21 (triceps + subscapular)- 0.008 (triceps + subscapular)^2^ - 1.7) and (0.783 (triceps + subscapular) +1.6), respectively. Body mass index (BMI) was calculated as weight in kilograms divided by squared height in meters (kg/m^2^). Age and gender specific BMI Z-scores were calculated (child value minus mean value for gender and age group divided by standard deviation for gender and age group) using a contemporary Danish reference population [22]. BMI Z-score-difference (ΔBMI) from birth to school age was calculated by subtracting the Z-score at birth from the Z-score at school age. A ΔBMI Z-score >0.67 was considered a significant change (catch up or catch down), as 0.67 SD represents the width of each centile band of standard growth charts (i.e. 2nd to 9th, 9th to 25th, 25th to 50 centiles etc.) [23]. Differences in ΔBMI Z-scores were evaluated as a categorical variable (+/− clinically significant change) by Chi square analysis and as a continuous variable by multiple linear regression analysis.

Differences in characteristics between the group of children with the QQ and the QR/RR genotypes were tested by Mann-Whitney U test (continuous data) or Fisher’s Exact test (categorical data with two categories) or Likelihood Ratio (categorical data with more than two categories).

Logarithmic transformation of the variables was used when necessary to approach a normal distribution of residuals and was required for paraoxonase 1 activity, abdominal circumference, sum of four skin folds, body fat percentages, CV_RR,_ and serum concentrations of leptin, insulin, and IGF-I.

Age- and gender-adjusted partial correlation analyses were used to investigate associations between paraoxonase 1 activity and outcome variables. Effects of prenatal pesticide exposure were analyzed by multiple linear regression analysis. First, interactions between the *PON*1 Q192R genotype (0 = QQ, 1 =  QR/RR) and prenatal pesticide exposure (0 = unexposed, 1 = medium or high exposure) were tested by including a product term in the models. Subsequently, analyses were run separately for the group of children with the QQ and QR/RR genotype as well as for the combined group of children. In the stratified analyses, two dummy variables, one for high and one for medium prenatal pesticide exposure, were used in the models with no exposure as reference. Maternal smoking in pregnancy was treated as a dichotomous variable. Due to the small sample size, social class was re-coded into three categories: groups 1–3, group 4 or group 5 and included in the models as two dummy variables with group 4 as reference as most families belonged to this group. Covariates were identified from *a priori* considerations of relevant factors that might influence the outcome variables. For anthropometric outcomes, age at examination, gender, social class and maternal smoking in pregnancy were considered as obligatory covariates. For ΔBMI Z-score, gestational age was included in the models. Blood pressure was correlated to body composition, and therefore BMI was included in the models. Metabolic biomarkers were analyzed both with and without BMI in the models. The results are presented as the mean difference, or the relative difference in percent for log-transformed outcomes, with 95% confidence intervals (95%CI). A *p*-value below 0.05 was considered statistically significant.

## Results

This paper present the results for 141 children for whom *PON1* genotypes were determined. The genotype frequencies for *PON1* Q192R were 56.7% QQ, 35.5% QR, and 7.8% RR corresponding to an R-allele frequency of 25.5%. The distribution approximated the Hardy-Weinberg equilibrium. QQ homozygous children had significantly lower paraoxonase 1 activity than the R-carriers (Table 1). Among the R-carriers, none had the 55 MM genotype compared to 25% of those with the QQ type. None of the other characteristics differed significantly between children with the QQ genotype and the QR/RR genotype. The *PON1* Q192R genotype explained 66% of the variance in the paraoxonase activity. No significant correlations between paraoxonase activity and any outcome variables were seen (data not shown).

**Table 1:** Characteristics of 141 children with genotype *PON1* 192QQ or QR/RR examined at age 6–11 years.

Outcome^ a^	*PON1* 192QQ	*PON1* 192QR/RR	*p*-value
No of boys/girls (% boys)	47/33 (58.8)	35/26 (57.4)	1.00
Prenatal pesticide exposure [N (%)]			0.87
No exposure	31 (38.8)	22 (36.1)	
Medium exposure	28 (35.0)	24 (39.3)	
High exposure	21 (26.3)	15 (24.6)	
New recruited controls [N (%)]	21 (26.3)	16 (26.2)	1.00
Age (years)	8.7 (6.7; 10.6)	8.3 (6.8; 10.6)	0.26
Height (cm)	134.2 (121.2; 145.5)	132.9 (116.1; 146.2)	0.45
Weight (kg)	29.7 (22.9; 39.5)	28.4 (19.9; 43.4)	0.50
Parental social class [N (%)]			0.29
1–3	28 (35.0)	14 (23.0)	
4	37 (46.3)	33 (47.1)	
5	15 (18.8)	14 (23)	
Birth weight (g)	3520 (2590; 4677)	3735 (2295; 4455)	0.25
Gestational age (days)	283 (255; 297)	282 (261; 296)	0.94
Maternal smoking in pregnancy [no. (%)]	19 (23.8)	13 (21.3)	0.84
Maternal alcohol consumption in pregnancy [no. (%)]	32 (40.0)	18 (30.5)	0.29
Mother of non-Danish origin	4 (5.0)	2 (3.3)	0.70
Paraoxonase activity (nmol/min/ml)	29.6 (18.5; 29.6)	58.5 (42.4; 72.1)	<0.001
*PON1* 55 [N (%)]			<0.001
LL	22 (27.5)	31 (51)	
ML	38 (47.5)	30 (49)	
MM	20 (25.0)	0	
*PON1* 108 [N (%)]			0.15
CC	19 (23.8)	21 (34.4)	
CT	46 (57.6)	25 (41.0)	
TT	15 (18.8)	15 (24.6)	
			

For continuous variables, data represent median (5;95 percentiles).

b Differences between groups were tested with Mann-Whitney U-Test (continuous data) or Fishers Exact Test (categorical data with two categories) or Likelihood Ratio (categorical data with more than two categories).

Significant interactions between *PON1* Q192R genotype and prenatal pesticide exposure were seen for all outcomes, except systolic blood pressure, CV_RR_, and non-fasting serum concentrations of insulin, both before and after adjusting for covariates (Table 2). This finding indicates a higher susceptibility towards prenatal pesticide exposure in individuals with the R-allele. In genotype stratified analysis, an exposure-related increase in abdominal circumference, skin fold thickness, body-fat percentage (Table 3), BMI Z-score, BMI Z-score difference from birth to school age (Table 4) and in systolic and diastolic blood pressure (Table 5) was seen in children with the R-allele. CV_RR_ tended to be lower in exposed than in the unexposed R-carriers but the difference was not statistically significant, possibly due to a large variation in the CV_RR_ data. Exclusion of three children with CV_RR_ above 20% and one child with CV_RR_ below 2% did not materially change the results. For QQ-homozygote children, none of the outcome variables was significantly affected by prenatal pesticide exposure.

**Table 2:** Crude and adjusted estimated effects (β, 95%CI) of prenatal pesticide exposure, and *PON1* Q192R genotype on anthropometric outcomes, blood pressure, and metabolic biomarkers at school age.

	Crude model		Adjusted model	
	β (95%CI)	*p*-value	β (95%CI)	*p*-value
**Abdominal circumference^a^ (cm)**				
*PON1* 192QR/RR (unexposed)	−6.30 (−11.32;−1.00)	0.02	−6.22 (−11.01; −1.18)	0.02
Pesticide exposure (*PON1* 192QQ)	−2.00 (−6.34; 2.54)	0.38	−2.26 (−6.40; 2.06)	0.30
Pesticide exposure (*PON1* 192QR/RR) (interaction)	10.09 (2.71; 18.01)	0.01	10.35 (3.34; 17.85)	0.004
**Sum of four skin folds^a^ (mm)**				
*PON1* 192QR/RR (unexposed)	−20.12 (−34.3; −2.87)	0.03	−21.81 (−35.24; −5.61)	0.01
Pesticide exposure (*PON1* 192QQ)	−5.88 (−19.88; 10.55)	0.46	−7.68 (−20.97; 7.85)	0.31
Pesticide exposure (*PON1* 192QR/RR) (interaction)	49.01 (16.4290.71)	0.002	52.94 (20.80; 93.64)	0.001
**Body fat percentages^a^ (%)**				
*PON1* 192QR/RR (unexposed)	−15.79 (−27.72; −1.90)	0.03	−16.44 (−28.05; −2.95)	0.02
Pesticide exposure (*PON1* 192QQ)	−4.86 (−16.10; 7.88)	0.44	−5.82 (−16.76; 6.56)	0.34
Pesticide exposure (*PON1* 192QR/RR) (interaction)	35.47 (11.73; 64.26)	0.002	37.00 (13.58; 65.24)	0.001
**BMI Z-score at school age^b^**				
*PON1* 192QR/RR (unexposed)	−0.70 (−1.36; −0.04)	0.04	−0.78 (−1.43; −0.13)	0.02
Pesticide exposure (*PON1* 192QQ)	−0.15 (−0.69; 0.39)	0.59	−0.26 (−0.80; 0.28)	0.34
Pesticide exposure (*PON1* 192QR/RR) (interaction)	1.30 (0.47; 2.13)	0.002	1.36 (0.54; 2.17)	0.001
Δ**BMI Z-score between school age and birth^c^**				
*PON1* 192QR/RR (unexposed)	−0.61 (−1.45; 0.23)	0.16	−0.53 (−1.30; 0.24)	0.18
Pesticide exposure (*PON1* 192QQ)	0.13 (−0.56; 0.83)	0.71	−0.07 (−0.70; 0.57)	0.83
Pesticide exposure (*PON1* 192QR/RR) (interaction)	1.43 (0.36; 2.49)	0.01	1.32 (0.35; 2.28)	0.01
**Systolic blood pressure^d^ (mmHg)**				
*PON1* 192QR/RR (unexposed)	−0.61 (−4.75; 3.54)	0.77	−0.30 (−4.53; 3.94)	0.89
Pesticide exposure (*PON1* 192QQ)	−0.67 (−4.09; 2.74)	0.70	−0.57 (−3.99; 2.86)	0.74
Pesticide exposure (*PON1* 192QR/RR) (interaction)	3.28 (−1.97; 8.53)	0.22	3.19 (−2.26; 8.64)	0.25
**Diastolic blood pressure^d^ (mmHg)** *PON1* 192QR/RR (unexposed)	−0.88 (−4.29; 2.53)	0.61	−0.50 (−4.00; 3.00)	0.78
Pesticide exposure (*PON1* 192QQ)	−1.45 (−4.26; 1.36)	0.31	−1.30 (−4.13; 1.53)	0.37
Pesticide exposure (*PON1* 192QR/RR) (interaction)	5.35 (1.03; 9.66)	0.02	4.89 (0.38; 9.40)	0.03
**CV_RR_^e^**				
*PON1* 192QR/RR (unexposed)	−8.7 (−35.9; 30.0)	0.61	−11.2 (−37.8; 26.7)	0.51
Pesticide exposure (*PON1* 192QQ)	6.1 (−20.7; 42.1)	0.69	7.2 (−19.8; 43.4)	0.63
Pesticide exposure (*PON1* 192QR/RR) (interaction)	−23.4 (−49.7; 16.4)	0.21	−22.4 (−49.0; 18.0)	0.23
**Leptin^f^ (ng/ml)**				
*PON1* 192QR/RR (unexposed)	−34.8 (−61.2; 9.3)	0.10	−33.9 (−60.4; 10.2)	0.11
Pesticide exposure (*PON1* 192QQ)	28.2 (−17.0; 98.0)	0.26	31.3 (−14.5; 101.6)	0.21
Pesticide exposure (*PON1* 192QR/RR) (interaction)	135.2 (20.8; 357.8)	0.01	141.7 (25.2; 366.7)	0.01
**Insulin^f^ (ng/ml)**				
*PON1* 192QR/RR (unexposed)	14.0 (−31.7; 90.4)	0.61	10.7 (−33.9; 85.4)	0.70
Pesticide exposure (*PON1* 192QQ)	31.6 (−14.7; 103.0)	0.21	30.6 (−15.4; 101.8)	0.23
Pesticide exposure (*PON1* 192QR/RR) (interaction)	11.7 (−41.8; 114.6)	0.74	15.4 (−40.1; 122.4)	0.67
**IGF-I^f^ (ng/ml)**				
*PON1* 192QR/RR (unexposed)	−21.3 (−36.2; −2.9)	0.03	−20.4 (−34.3; −3.5)	0.02
Pesticide exposure (*PON1* 192QQ)	−14.8 (−28.3; 1.3)	0.07	−13.0 (−25.7; 1.8)	0.08
Pesticide exposure (*PON1* 192QR/RR) (interaction)	48.9 (14.2; 94.2)	0.004	50.1 (17.9; 91.1)	0.001
**IGFBP3^f^ (ng/ml)**				
*PON1* 192QR/RR (unexposed)	−273.9 (−636.2; 88.5)	0.14	−253.6 (−605.1; 98.0)	0.16
Pesticide exposure (*PON1* 192QQ)	−78.2 (−376.5; 220.1)	0.61	−52.8 (−341.4; 235.9)	0.72
Pesticide exposure (*PON1* 192QR/RR) (interaction)	467.4 (0.1; 924.7)	0.05	469.9 (27.3; 912.4)	0.04

Β expresses the mean differences for BMI Z-score at school age, ΔBMI Z-score between school age and birth, systolic and diastolic blood pressure, and IGFBP3 and the relative difference (in percent) for ln-transformed outcomes (abdominal circumference, sum of four skin folds, body fat percentages, coefficient of variation for heart rate (CV_RR_), leptin, insulin, and IGF-I).

Adjusted model included ^a^gender, age at examination, social class, and maternal smoking in pregnancy; ^b^social class, and maternal smoking in pregnancy; ^c^ gestational age, social class, and maternal smoking in pregnancy; ^d^gender, age at examination, maternal smoking in pregnancy, and BMI; ^e^gender, age at examination, and maternal smoking in pregnancy; ^f^gender and age at examination.

**Table 3:** Adjusted values^a^ for anthropometric outcomes at school age in children in relation to prenatal pesticide exposure and *PON1* Q192R genotype.

	Adjusted geometric mean (95% CI)		
	All children	*PON1* 192QQ	*PON1* 192QR/RR
**Abdominal circumference (cm)**			
No exposure	62.3 (60.6; 63.9)	63.2 (61.0; 65.4)	59.2 (56.8; 61.7)##
Medium exposure	63.2 (61.6; 64.8)	61.0 (58.8; 63.3)	62.4 (60.0; 64.8)
High exposure	63.8 (61.9; 65.8)	62.7 (60.1; 65.4)	65.9 (62.7; 69.3) **
**Sum of four skin folds (mm)**			
No exposure	40 (36; 44)	43 (38; 49)	34 (30; 39)##
Medium exposure	44 (40; 48)	36 (32; 42)	44 (39; 50) #**
High exposure	46 (41; 52)	44 (38; 51)	52 (44; 62) ***
**Body fat percentages- (%)**			
No exposure	18.1 (16.8; 19.5)	19.1 (17.2; 21.1)	16.0 (14.4; 17.9)#
Medium exposure	19.1 (17.8; 20.6)	16.8 (15.1; 18.7)	19.5 (17.5; 21.6)#**
High exposure	20.4 (18.7; 22.3) *	19.6 (17.4; 22.2)	22.3 (19.6; 25.5) ***

a Results from linear regression model adjusted for gender, age at examination, social class, and maternal smoking in pregnancy. The outcome variables were ln-transformed.

* p≤0.05

** p≤0.01

*** p≤0.001 compared to unexposed.

# p≤0.05

## p≤0.01 compared to *PON1* 192QQ in the same exposure group.

**Table 4:** Multiple regression analysis of prenatal pesticide exposure, maternal smoking and social class as predictors for BMI Z-scores at school age, and difference in BMI Z-scores between birth and school age in children with the *PON1* 192 QQ or QR/RR genotype.

	*PON1* 192QQ		*PON1* 192QR/RR	
	β (95% CI)	*p*-value	β (95% CI)	*p*-value
**BMI Z-score at school age**				
Social class 5	0.49 (−0.24; 1.22)	0.19	0.07 (−0.69; 0.82)	0.86
Social class 1–3	−0.43 (−1.01; 0.16)	0.15	−0.19 (−0.96; 0.59)	0.64
Maternal smoking in pregnancy	0.10 (−0.54; 0.74)	0.77	0.77 (0.021; 1.52)	0.04
Maternal pesticide exposure medium level	−0.45 (−1.06; 0.15)	0.14	0.79 (0.09; 1.50)	0.03
Maternal pesticide exposure high level	−0.07 (−0.73; 0.58)	0.82	1.57 (0.76; 2.37)	<0.001
Δ**BMI Z-score between school age and birth**				
Gestational age (days)	−0.04 (−0.06; −0.01)	0.003	−0.07 (−0.10; −0.03)	<0.001
Social class 5	0.77 (−0.12; 1.66)	0.09	0.21 (−0.68; 1.09)	0.64
Social class 1–3	−0.77 (−1.50; −0.04)	0.04	−0.29 (−1.19; 0.61)	0.52
Maternal smoking in pregnancy	0.33 (−0.44; 1.11)	0.39	0.82 (−0.07; 1.70)	0.07
Maternal pesticide exposure medium level	−0.35 (−1.08; 0.39)	0.35	1.13 (0.28; 1.99)	0.01
Maternal pesticide exposure high level	0.15 (−0.66; 0.97)	0.71	1.29 (0.36; 2.21)	0.007

**Table 5:** Adjusted values^a^ for systolic and diastolic blood pressure and coefficient of variation for heart rate (CV_RR_) at school age in relation to prenatal pesticide exposure and *PON1* Q192R genotype.

	Adjusted mean/geometric mean (95% CI)		
	**All children**	***PON1*** ** 192QQ**	***PON1*** ** 192QR/RR**
**Systolic blood pressure (mmHg)**			
No exposure	100.6 (98.7; 102.6)	100.1 (97.4; 102.7)	99.1 (95.6; 102.7)
Medium exposure	100.1 (98.2; 102.0)	99.4 (96.6; 102.3)	99.6 (96.6; 102.5)
High exposure	101.7 (99.4; 104.0)	99.7 (96.4; 103.0)	108.4 (104.1; 112.7) ## ***
**Diastolic blood pressure (mmHg)**			
No exposure	58.9 (57.2; 60.6)	58.2 (55.9; 60.4)	57.4 (54.8; 60.0)
Medium exposure	59.2 (57.5; 60.8)	58.1 (55.7; 60.5)	61.1 (58.7; 63.4) #*
High exposure	57.8 (55.8; 59.7)	55.5 (52.8; 58.3)	62.0 (58.6; 65.5) #*
**CV_RR_** b			
No exposure	7.4 (6.3; 8.7)	8.2 (6.3; 10.7)	7.4 (5.8; 9.5)
Medium exposure	7.4 (6.5; 8.4)	8.8 (7.1; 10.8)	6.1 (4.9; 7.5) ##
High exposure	7.5 (6.3; 8.8)	8.7 (6.7; 11.2)	6.1 (4.7; 8.0) #

a Results from linear regression models adjusted for gender, age at examination, maternal smoking in pregnancy, and for systolic and diastolic blood pressure also BMI. CV_RR_ was ln-transformed.

* p≤0.05,

*** p≤0.001 compared to unexposed.

# p≤0.05,

## p≤0.01 compared to *PON1* 192QQ in the same exposure group.

b Due to technical problems with the Heart Rate Monitor, CV_RR_ was only obtained for 98 (14 QQ and 14 QR/RR unexposed, 23 QQ and 19 QR/RR medium exposed, and 16 QQ and 12 QR/RR high exposed) of the 141 children for whom *PON1* genotype was determined.

The mean birth weight was significantly lower for exposed than unexposed children (3521 g versus 3677 g, p = 0.04). This association was seen for both QQ homozygotes and R-carriers although it did not reach significance for the genotypes separately (data not shown). Among the R-carriers, 53.8% of the exposed children had an increase in ΔBMI Z-score of more than 0.67 compared to 13.6% among the unexposed (p  = 0.003). For the QQ-homozygotes, the proportion of children with ΔBMI Z-score above 0.67 was similar for exposed and unexposed, 32.7% and 29.0%, respectively (p = 0.81). The low percentage for the group of unexposed R-carriers was due to negative BMI Z-scores at school age and negative difference in BMI Z-scores from birth to school age. Hence, this group of children was leaner than the reference population and also the unexposed QQ-homozygotes. The same pattern was seen for abdominal circumference, sum of four skin folds, body fat percentage, and serum concentration of IGF-I (Tables 2, 3, 4, 5, 6). Systolic and diastolic blood pressure, CV_RR_, and serum concentration of leptin did not differ between unexposed R-carriers and QQ-homozygotes (Table 5 and 6). In R-carriers, non-fasting serum concentrations of leptin and IGF-I were increased after prenatal pesticide exposure in an exposure dependent manner (Table 6). After adjusting for BMI, the effect on leptin, but not IGF-I, remained significant (data not shown). For insulin and IGFBP-3, the results were less consistent.

**Table 6:** Adjusted values^a^ for non-fasting serum concentrations of leptin, insulin, IGF1, and IGFBP3 at school age in relation to prenatal pesticide exposure and *PON1* Q192R genotype.

	Adjusted mean/geometric mean (95% CI)		
	**All children**	***PON1*** ** 192QQ**	***PON1*** ** 192QR/RR**
**Leptin (ng/ml)**			
No exposure	2.06 (1.60; 2.66)	2.48 (1.73; 3.57)	1.57 (1.11; 2.22)
Medium exposure	3.67 (2.81; 4.79)**	2.92 (1.95; 4.37)	4.41 (3.13; 6.22) ***
High exposure	4.55 (3.26; 6.36)***	3.77 (2.32; 6.13)	6.47 (4.07; 10.29) ***
**Insulin (ng/ml)**			
No exposure	0.42 (0.33; 0.54)	0.40 (0.29; 0.54)	0.47 (0.30; 0.71)
Medium exposure	0.67 (0.52; 0.86)**	0.58 (0.42; 0.81)	0.90 (0.61; 1.33)*
High exposure	0.46 (0.33; 0.64)	0.45 (0.30; 0.67)	0.35 (0.20; 0.60)
**IGF-I (ng/ml)**			
No exposure	143.9 (131.0; 158.1)	158.7 (139.8; 180.1)	125.1 (108.2; 144.6)##
Medium exposure	139.0 (126.5; 152.7)	127.0 (111.2; 145.1)*	155.4 (135.2; 178.6) #*
High exposure	163.1 (145.4; 183.0)	154.8 (132.8; 180.5)	173.0 (144.6; 206.9)**
**IGFBP3 (ng/ml)**			
No exposure	3339 (3169; 351)	3341 (3212; 3670)	3169 (2897; 3441)
Medium exposure	3369 (3198; 3540)	3209 (2971; 3447)	3586 (3323; 3849) #*
High exposure	3592 (3384; 3802)	3615 (3341; 3890)	3558 (3219; 3897)

a Results from linear regression models adjusted for gender and age. Leptin, insulin, and IGF-I were ln-transformed.

* p≤0.05,

** p≤0.01,

*** p≤0.001 compared to unexposed.

# p≤0.05,

## p≤0.01 compared to *PON1* 192QQ unexposed.

Within the group of children with the QQ genotype, those with the 55MM genotype had higher birth weight than those with the 55LL or 55 ML type (data not shown) but none of the outcome variables measured at school age differed between the *PON1* L55M genotypes. Including the *PON1* L55M genotypes in the regression analysis (modelled as 0 (MM), 1 (ML), and 2 (LL)) did not change the results.

## Discussion

Despite the small sample size, our study found indications of a gene-environment interaction between prenatal pesticide exposure and the *PON1* Q192R genotype that might affect the risk of obesity and related diseases later in life. Prenatally pesticide-exposed children carrying the R-allele had higher abdominal circumference, body fat content, BMI Z-scores, systolic and diastolic blood pressure, and serum concentrations of leptin and IGF-I at school age than did unexposed children. For QQ-homozygous children, none of these variables were significantly affected by prenatal pesticide exposure. The results indicate that R-carriers may be especially susceptible towards developmental disturbances after prenatal pesticide exposure.

An important strength of this study is the longitudinal design that minimizes exposure misclassification and bias. The classification of the mothers as high, medium or not exposed was done independently by two toxicologists with special expertise in working conditions in greenhouse horticultures at enrolment early in pregnancy [13], [14] and hence completely independent of subsequent examination outcomes for the children.

The unexposed group was extended at the follow-up examination to provide better comparison data. None of the mothers of these children had worked in greenhouses or other occupations where pesticides were used during pregnancy. They were distributed evenly between the group of children with the R-allele and the QQ homozygotes, and, therefore, did not affect the differences observed in response to prenatal pesticide exposure between the two genotypes. In addition, all examinations of the children were done blinded to the exposure information and by the same paediatrician using standardized procedures. Genotyping of *PON1* and analyses of serum samples were performed blinded to both exposure information and examination outcomes.

The small number of exposed and unexposed children within each of the two *PON1* genotypes is a limitation of the study, because it decreases the power of the study and diminishes the possibility of detecting exposure related effects. Nonetheless, if detectable in a small study like ours, the associations may be important. An additional limitation is that we conducted many statistical analyses and chose not to adjust for multiple comparisons, although they may increase the likelihood of spurious associations. However, several of the outcomes are interrelated and the observed associations between prenatal pesticide exposure and effects in the group of children with the R-allele were very robust being significant both before and after adjusting for covariates. In addition, the magnitudes of the effects were related to the exposure level which again supports a causal association. Finally, the distribution of known risk factors such as social class and maternal smoking in pregnancy did not differ between the children with the R-allele and the QQ-homozygotes.

Another possible limitation of the study is that the *PON1* genotypes of the mothers were not determined. The maternal genotype and related ability to metabolize pesticides might be important for protection of the foetus against exposure. However, only 11 children (6 unexposed and 5 exposed) were homozygous for the R-allele, and for the heterozygous children approximately half of the mothers would be assumed to be QQ homozygotes. Therefore, it is unlikely that the observed associations would be explained by the maternal genotype.

In a previous study, women who worked in floricultures in Mexico and had the RR genotype had higher risk of having children with low birth weight than women with the QQ or QR genotype [24]. In another study, maternal organophosphate exposure during pregnancy was associated with reduced head circumference and head size of the children at birth if the mothers had a low PON1 concentration but no association was seen between neither maternal nor child *PON1* genotype and head circumference or birth weight or birth length [25]. However, at 12 months of age, negative associations between prenatal organophosphate exposure and cognitive development were seen if the mothers had the QR/RR genotype, although later in childhood children of mothers with the QQ-genotype appeared more affected [26]. In a recent study, shorter gestation and smaller head circumference at birth were associated to low infant, but not maternal, PON1 enzyme activity [27]. This association was independent of maternal organophosphate exposure in pregnancy but for infants with low activity (and *PON1* -108TT and *PON1* 192QQ genotype) an association between maternal organophosphate exposure in pregnancy and shorter gestational age was seen. Although, the R-allele seems to provide better protection than the Q-allele toward toxic effects of certain organophosphates such as chlorpyrifos [8], [28], the capacity of *PON1* allozymes to protect LDL from oxidation might be different, and even the reverse, of the paraoxonase activity, with the lowest capacity for the R-allozyme [1], [3].

A weak association between the R-allele and increased CVD risk has been confirmed in most case-control studies, and is supported by meta-analyses [2], [9], [29]–[33]. Results from studies on associations between *PON1* R192Q genotype and risk markers for CVD in healthy populations are inconsistent, as some found no association [34], [35] or a higher frequency of the R-allele among obese (BMI ≥30 kg/m^2^) compared to normal-weight pre-menopausal women [36], while increased blood pressure was associated with the R-allele in women above 60 years [30], and in a rural population in Greece [37]. In general, these studies do not include information about environmental exposures, and discrepancies might be due to unidentified gene-environment or gene-gene interactions. In a recent study the combination of low serum HDL concentration and the RR genotype markedly increased the risk of CVD [38]. This interaction is also supported by the results from our study where no marked difference in the risk profile between unexposed R-carriers and QQ-homozygotes was seen. In fact, the R-carriers were leaner than the QQ homozygotes at school age. However, the combination of the R-allele and prenatal pesticide exposure had a pronounced effect on the risk profiles.

In our study, pesticide-exposed children with the R-allele had a higher blood pressure and a lower CV_RR_, although only the former reached statistical significance. Increased blood pressure was also reported for children, whose mothers were occupationally exposed to pesticides during pregnancy in Ecuador [39]. The *PON1* genotype was not determined in these studies, but the R-allele frequency is likely to be higher than in our study, since an R-allele frequency of 40 to 60% has been reported for many populations in Latin and South America [24]. Due to maturation of the cardiac autonomic activity, CV_RR_ increases during gestation and early postnatal life followed by a decline [40]. Prenatal exposure to the neurotoxicant methyl mercury caused decreased CV_RR_ in children at 7 and 14 years of age [41].

Children prenatally exposed to pesticides had lower birth weight than unexposed children as previously reported for the children in this study [12], and also reported in several other studies [42]–[45]. For children with the R-allele, this was followed by accelerated body fat accumulation until school age. Low birth weight followed by catch-up growth and body fat accumulation during childhood is associated with increased risk of obesity, insulin and leptin resistance and CVD later in life [46]–[48,48]. There is increasing evidence that prenatal and early postnatal environmental factors might influence the risk [49], [50]. In addition, prenatal exposure to low doses of some endocrine disrupting chemicals has been demonstrated to cause obesity in rodents [51]–[53], and epidemiological studies also have linked exposure to some of these chemicals to obesity and metabolic syndrome in humans [54]–[56]. The mechanisms behind these associations are not fully understood, but might include epigenetic changes that affect gene expression [57], [58] and fetal programming of energy balance [59], perhaps including disturbance of the hypothalamic-pituitary-adrenal axis [60]. In rats, low doses of chlorpyriphos during development caused excess weight gain and leptin and insulin dysregulation later in life [61], [62] and neonatal parathion exposure disrupted the production of adipokines, including leptin, that regulate appetite by communicating metabolic status between adipose tissues and the brain [63]. Hence, these effects resemble those seen in the exposed children expressing the R-allele.

In the present study, the mothers were exposed early in their pregnancies to a variety of different pesticides including organophosphates and several fungicides with known endocrine disrupting properties [64], [65]. All of the substances had been approved by the national regulatory agency in accordance with European Union legislation. Due to the complex exposure setting, it was not possible to identify specific pesticides as responsible for the effects. They may be due to the combined exposure to several pesticides and maybe also to other ingredients added to the pesticide formulations. For most of the exposed mothers, organophosphates had been used occasionally in the working areas, but only very few mothers had been involved directly in their application. Whether PON1 can detoxify other types of pesticides than organophosphates has, to our knowledge, not been investigated.

In conclusion, this study indicates a gene-environment interaction between prenatal pesticide exposure and *PON1* gene heterogeneities that affects cardiovascular risk markers already thought to be associated with the R-allele, as such [2]. Individuals with the R allele in the 192 position seem particularly susceptible, and the combination of this genotype and prenatal pesticide exposure significantly stimulated fat accumulation from birth to school age, caused higher blood pressure and enhanced serum concentration of leptin and IGF-I. These effects are all related to increased risk for development of metabolic syndrome and CVD later in life. The results also illustrate that a susceptible subgroup of a population could be affected, although effects may not be evident in the entire population.

## References

[1] Mackness B, Mackness MI, Arrol S, Turkie W, Durrington PN (1998). Effect of the human serum paraoxonase 55 and 192 genetic polymorphisms on the protection by high density lipoprotein against low density lipoprotein oxidative modification.. FEBS Lett.

[2] Durrington PN, Mackness B, Mackness MI (2001). Paraoxonase and atherosclerosis.. Arterioscler Thromb Vasc Biol.

[3] Aviram M, Rosenblat M, Bisgaier CL, Newton RS, Primo-Parmo SL (1998). Paraoxonase inhibits high-density lipoprotein oxidation and preserves its functions. A possible peroxidative role for paraoxonase.. J Clin Invest.

[4] Mackness B, Durrington P, Povey A, Thomson S, Dippnall M (2003). Paraoxonase and susceptibility to organophosphorus poisoning in farmers dipping sheep.. Pharmacogenetics.

[5] Costa LG, Cole TB, Furlong CE (2003). Polymorphisms of paraoxonase (PON1) and their significance in clinical toxicology of organophosphates.. J Toxicol Clin Toxicol.

[6] Leviev I, James RW (2000). Promoter polymorphisms of human paraoxonase PON1 gene and serum paraoxonase activities and concentrations.. Arterioscler Thromb Vasc Biol.

[7] Brophy VH, Hastings MD, Clendenning JB, Richter RJ, Jarvik GP (2001). Polymorphisms in the human paraoxonase (PON1) promoter.. Pharmacogenetics.

[8] Davies HG, Richter RJ, Keifer M, Broomfield CA, Sowalla J (1996). The effect of the human serum paraoxonase polymorphism is reversed with diazoxon, soman and sarin.. Nat Genet.

[9] Seo D, Goldschmidt-Clermont P (2009). The paraoxonase gene family and atherosclerosis.. Curr Atheroscler Rep.

[10] Mackness B, Mackness MI, Durrington PN, Arrol S, Evans AE (2000). Paraoxonase activity in two healthy populations with differing rates of coronary heart disease.. Eur J Clin Invest.

[11] Shih DM, Gu L, Xia YR, Navab M, Li WF (1998). Mice lacking serum paraoxonase are susceptible to organophosphate toxicity and atherosclerosis.. Nature.

[12] Wohlfahrt-Veje C, Main KM, Schmidt IM, Boas M, Jensen TK (2011). Lower birth weight and increased body fat at school age in children prenatally exposed to modern pesticides: A prospective study.. Environ Health.

[13] Andersen HR, Schmidt IM, Grandjean P, Jensen TK, Budtz-Jorgensen E (2008). Impaired Reproductive Development in Sons of Women Occupationally Exposed to Pesticides during Pregnancy.. Environ Health Perspect.

[14] Andersen HR, Nielsen F, Nielsen JB, Kjaerstad MB, Baelum J (2007). Xeno-oestrogenic activity in serum as marker of occupational pesticide exposure.. Occup Environ Med.

[15] Hansen, EJ (1978). Distribution of living conditions..

[16] Hansen, EJ (1986). Danskernes levevilkår 1986 sammenholdt med 1976..

[17] Rodriguez G, Moreno LA, Blay MG, Blay VA, Fleta J (2005). Body fat measurement in adolescents: comparison of skinfold thickness equations with dual-energy X-ray absorptiometry.. Eur J Clin Nutr.

[18] Christiansen L, Bathum L, Frederiksen H, Christensen K (2004). Paraoxonase 1 polymorphisms and survival.. Eur J Hum Genet.

[19] Dalgard C, Christiansen L, Jonung T, Mackness MI, de Maat MP (2007). No influence of increased intake of orange and blackcurrant juices and dietary amounts of vitamin E on paraoxonase-1 activity in patients with peripheral arterial disease.. Eur J Nutr.

[20] Mackness MI, Harty D, Bhatnagar D, Winocour PH, Arrol S (1991). Serum paraoxonase activity in familial hypercholesterolaemia and insulin-dependent diabetes mellitus.. Atherosclerosis.

[21] Slaughter MH, Lohman TG, Boileau RA, Horswill CA, Stillman RJ (1988). Skinfold equations for estimation of body fatness in children and youth.. Hum Biol.

[22] Nysom K, Molgaard C, Hutchings B, Michaelsen KF (2001). Body mass index of 0 to 45-y-old Danes: reference values and comparison with published European reference values.. Int J Obes Relat Metab Disord.

[23] Ong KK, Ahmed ML, Emmett PM, Preece MA, Dunger DB (2000). Association between postnatal catch-up growth and obesity in childhood: prospective cohort study.. BMJ.

[24] Moreno-Banda G, Blanco-Munoz J, Lacasana M, Rothenberg SJ, Aguilar-Garduno C (2009). Maternal exposure to floricultural work during pregnancy, PON1 Q192R polymorphisms and the risk of low birth weight.. Sci Total Environ.

[25] Berkowitz GS, Wetmur JG, Birman-Deych E, Obel J, Lapinski RH (2004). In utero pesticide exposure, maternal paraoxonase activity, and head circumference.. Environ Health Perspect.

[26] Engel SM, Wetmur J, Chen J, Zhu C, Barr DB (2011). Prenatal Exposure to Organophosphates, Paraoxonase 1, and Cognitive Development in Childhood.. Environ Health Perspect.

[27] Harley KG, Huen K, Schall RA, Holland NT, Bradman A (2011). Association of organophosphate pesticide exposure and paraoxonase with birth outcome in Mexican-American women.. PLoS ONE.

[28] Li WF, Costa LG, Richter RJ, Hagen T, Shih DM (2000). Catalytic efficiency determines the in-vivo efficacy of PON1 for detoxifying organophosphorus compounds.. Pharmacogenetics.

[29] Wheeler JG, Keavney BD, Watkins H, Collins R, Danesh J (2004). Four paraoxonase gene polymorphisms in 11212 cases of coronary heart disease and 12786 controls: meta-analysis of 43 studies.. Lancet.

[30] Lawlor DA, Day IN, Gaunt TR, Hinks LJ, Briggs PJ (2004). The association of the PON1 Q192R polymorphism with coronary heart disease: findings from the British Women’s Heart and Health cohort study and a meta-analysis.. BMC Genet.

[31] Banerjee I (2010). Relationship between Paraoxonase 1 (PON1) gene polymorphisms and susceptibility of stroke: a meta-analysis.. Eur J Epidemiol.

[32] Dahabreh IJ, Kitsios GD, Kent DM, Trikalinos TA (2010). Paraoxonase 1 polymorphisms and ischemic stroke risk: A systematic review and meta-analysis.. Genet Med.

[33] Wang M, Lang X, Zou L, Huang S, Xu Z (2011). Four genetic polymorphisms of paraoxonase gene and risk of coronary heart disease: a meta-analysis based on 88 case-control studies.. Atherosclerosis.

[34] Thyagarajan B, Jacobs DR, Carr JJ, Alozie O, Steffes MW (2008). Factors associated with paraoxonase genotypes and activity in a diverse, young, healthy population: the Coronary Artery Risk Development in Young Adults (CARDIA) study.. Clin Chem.

[35] Manresa JM, Zamora A, Tomas M, Senti M, Fito M (2006). Relationship of classical and non-classical risk factors with genetic variants relevant to coronary heart disease.. Eur J Cardiovasc Prev Rehabil.

[36] Veiga L, Silva-Nunes J, Melao A, Oliveira A, Duarte L (2011). Q192R polymorphism of the paraoxonase-1 gene as a risk factor for obesity in Portuguese women.. Eur J Endocrinol.

[37] Tsatsakis AM, Zafiropoulos A, Tzatzarakis MN, Tzanakakis GN, Kafatos A (2009). Relation of PON1 and CYP1A1 genetic polymorphisms to clinical findings in a cross-sectional study of a Greek rural population professionally exposed to pesticides.. Toxicol Lett.

[38] Mendonca MI, Dos Reis RP, Freitas AI, Pereira A, Sousa AC (2010). Interaction of paraoxonase-192 polymorphism with low HDL-cholesterol in coronary artery disease risk.. Rev Port Cardiol.

[39] Harari R, Julvez J, Murata K, Barr D, Bellinger DC (2010). Neurobehavioral deficits and increased blood pressure in school-age children prenatally exposed to pesticides..

[40] van Ravenswaaij-Arts CM, Kollee LA, Hopman JC, Stoelinga GB, van Geijn HP (1993). Heart rate variability.. Ann Intern Med.

[41] Grandjean P, Murata K, Budtz-Jorgensen E, Weihe P (2004). Cardiac autonomic activity in methylmercury neurotoxicity: 14-year follow-up of a Faroese birth cohort.. J Pediatr.

[42] Dabrowski S, Hanke W, Polanska K, Makowiec-Dabrowska T, Sobala W (2003). Pesticide exposure and birthweight: an epidemiological study in Central Poland.. Int J Occup Med Environ Health.

[43] Hanke W, Romitti P, Fuortes L, Sobala W, Mikulski M (2003). The use of pesticides in a Polish rural population and its effect on birth weight.. Int Arch Occup Environ Health.

[44] Levario-Carrillo M, Amato D, Ostrosky-Wegman P, Gonzalez-Horta C, Corona Y (2004). Relation between pesticide exposure and intrauterine growth retardation..

[45] Whyatt RM, Rauh V, Barr DB, Camann DE, Andrews HF (2004). Prenatal insecticide exposures and birth weight and length among an urban minority cohort.. Environ Health Perspect.

[46] Ong KK (2010). Early Determinants of Obesity.. Endocr Dev.

[47] Baird J, Fisher D, Lucas P, Kleijnen J, Roberts H (2005). Being big or growing fast: systematic review of size and growth in infancy and later obesity.. BMJ.

[48] Dulloo AG (2008). Thrifty energy metabolism in catch-up growth trajectories to insulin and leptin resistance.. Best Pract Res Clin Endocrinol Metab.

[49] Barker DJ (2004). The developmental origins of chronic adult disease.. Acta.

[50] Langley-Evans SC (2006). Developmental programming of health and disease.. Proc Nutr Soc.

[51] Grun F, Blumberg B (2009). Endocrine disrupters as obesogens.. Mol Cell Endocrinol.

[52] Heindel JJ, vom Saal FS (2009). Role of nutrition and environmental endocrine disrupting chemicals during the perinatal period on the aetiology of obesity.. Mol Cell Endocrinol.

[53] Newbold RR (2010). Impact of environmental endocrine disrupting chemicals on the development of obesity.. Hormones (Athens ).

[54] Vasiliu O, Cameron L, Gardiner J, Deguire P, Karmaus W (2006). Polybrominated biphenyls, polychlorinated biphenyls, body weight, and incidence of adult-onset diabetes mellitus.. Epidemiology.

[55] Everett CJ, Frithsen IL, Diaz VA, Koopman RJ, Simpson WM (2007). Association of a polychlorinated dibenzo-p-dioxin, a polychlorinated biphenyl, and DDT with diabetes in the 1999–2002 National Health and Nutrition Examination Survey.. Environ Res.

[56] Smink A, Ribas-Fito N, Garcia R, Torrent M, Mendez MA (2008). Exposure to hexachlorobenzene during pregnancy increases the risk of overweight in children aged 6 years.. Acta Paediatr.

[57] Jirtle RL, Skinner MK (2007). Environmental epigenomics and disease susceptibility.. Nat Rev Genet.

[58] Dolinoy DC, Weidman JR, Jirtle RL (2007). Epigenetic gene regulation: linking early developmental environment to adult disease.. Reprod Toxicol.

[59] Bourguignon JP, Rasier G, Lebrethon MC, Gerard A, Naveau E (2010). Neuroendocrine disruption of pubertal timing and interactions between homeostasis of reproduction and energy balance.. Mol Cell Endocrinol.

[60] Honour JW, Jones R, Leary S, Golding J, Ong KK (2007). Relationships of urinary adrenal steroids at age 8 years with birth weight, postnatal growth, blood pressure, and glucose metabolism.. J Clin Endocrinol Metab.

[61] Lassiter TL, Brimijoin S (2008). Rats gain excess weight after developmental exposure to the organophosphorothionate pesticide, chlorpyrifos.. Neurotoxicol Teratol.

[62] Slotkin TA, Brown KK, Seidler FJ (2005). Developmental exposure of rats to chlorpyrifos elicits sex-selective hyperlipidemia and hyperinsulinemia in adulthood.. Environ Health Perspect.

[63] Lassiter TL, Ryde IT, Levin ED, Seidler FJ, Slotkin TA (2010). Neonatal exposure to parathion alters lipid metabolism in adulthood: Interactions with dietary fat intake and implications for neurodevelopmental deficits.. Brain Res Bull.

[64] Andersen HR, Vinggaard AM, Rasmussen TH, Gjermandsen IM, Bonefeld-Jorgensen EC (2002). Effects of currently used pesticides in assays for estrogenicity, androgenicity, and aromatase activity in vitro.. Toxicol Appl Pharmacol.

[65] Orton F, Rosivatz E, Scholze M, Kortenkamp A (2011). Widely Used Pesticides with Previously Unknown Endocrine Activity Revealed as in Vitro Anti-Androgens.. Environ Health Perspect.

